# Internet Gaming Disorder, Sleep Quality, and Gaming Time Among High School and University Students in Saudi Arabia

**DOI:** 10.3390/healthcare14101348

**Published:** 2026-05-14

**Authors:** Emadeldin M. Elsokkary, Jehad A. Aldali, Mohammed B. AlQarni, Yazeed A. AlAhmari, Almuthanna S. Alghamdi, Dari N. Almodara, Abdulelah K. Alfandi

**Affiliations:** 1 Department of Psychology, College of Social Sciences, Imam Mohammad Ibn Saud Islamic University (IMSIU), Riyadh 13317, Saudi Arabia; 2 Department of Pathology, College of Medicine, Imam Mohammad Ibn Saud Islamic University (IMSIU), Riyadh 13317, Saudi Arabia; jaaldali@imamu.edu.sa; 3 College of Medicine, Imam Mohammad Ibn Saud Islamic University (IMSIU), Riyadh 13317, Saudi Arabia; 445013303@sm.imamu.edu.sa (M.B.A.); yazeed12309yy@gmail.com (Y.A.A.); almuthanna0@gmail.com (A.S.A.); abdulellah642642642@gmail.com (A.K.A.)

**Keywords:** Internet Gaming Disorder (IGD), sleep quality, Pittsburgh Sleep Quality Index (PSQI), daily gaming time, students

## Abstract

**Background:** Internet Gaming Disorder (IGD) has been linked to sleep disturbances, yet evidence from Saudi students is limited. **Objective:** We examined IGD symptom burden and its associations with daily gaming time and sleep quality among high school and university students in Riyadh, Saudi Arabia. **Methods:** In a cross-sectional online survey, participants completed the applicable consent process and were screened for gaming. Gamers completed a DSM-5–based IGD checklist and the Pittsburgh Sleep Quality Index (PSQI). IGD symptom count was analyzed using negative binomial regression (IRR), adjusting for sociodemographic and academic covariates and then adding PSQI total score. Results: Of invited students (N = 534), (n = 408) were gamers. Among those with complete PSQI totals (n = 352), the mean PSQI was (7.49) (SD = 3.46), and poor sleep (PSQI > 5) affected about two-thirds. Longer daily gaming time was significantly associated with higher expected IGD symptom counts. After adjustment, higher PSQI total score remained significantly associated with greater symptom burden (Adjusted IRR = 1.049 per 1-point increase), while demographic variables and academic-performance score were not significant. **Conclusions:** IGD symptom burden and poor sleep were common among student gamers in Riyadh. Longer daily gaming time and poorer sleep quality were associated with higher IGD symptom counts. These findings may inform prevention efforts related to gaming habits and sleep hygiene; however, longitudinal studies are needed to clarify temporal directionality and determine whether these associations reflect causal pathways.

## 1. Introduction

Life is influenced by multiple factors that may support or hinder individual growth and success [1]. Digital technologies such as smartphones [2] and social media platforms [3], alongside interactive digital entertainment—particularly video games—have become increasingly prominent in daily life. Video game use has increased in recent years, and gaming may offer educational and social benefits by supporting cognitive skills, strategic thinking, and teamwork [4]. However, excessive engagement with electronic media, including gaming, may be associated with adverse outcomes such as addictive behaviors [5].

The prevalence of problematic gaming and gaming-related difficulties appears to vary across countries, populations, and measurement approaches. In Saudi Arabia, Alrahili et al. reported a prevalence of 62% in a sample of 393 children and adolescents, whereas lower estimates have been reported in other Arab settings, including 5% in Jordan, 6% in Syria, and 7.8% in Kuwait [6,7]. Half of the studies included in the European review were conducted in Southern Europe (n = 10; 53%), with Spain contributing the largest number of studies (n = 7), followed by Italy (n = 2) and Greece (n = 1), whereas 42% of the studies (n = 8) were conducted in Western Europe, with Germany contributing four studies and France also represented; Denmark was the only Northern European country represented in the review 5% [8]. In the United States, Wan and Chiou (2007), as cited by the American Medical Association, reported that 90% of American adolescents play digital games and that 15% may show addictive patterns [9]. In Southeast Asia, a meta-analysis reported a pooled prevalence of 20.0% for Internet addiction and gaming disorders [10].

Previous studies have examined the associations of video game use with sleep quality among medical students [11], academic performance [12], and mental health outcomes such as anxiety, depression, and attention deficit hyperactivity disorder (ADHD) among children and adolescents in Saudi Arabia [7]. In recent years, the role of video games in students’ lives has changed noticeably, becoming part of the everyday routine for many university students and adolescents. This shift has raised concerns among teachers and parents about whether gaming may be associated with students’ academic performance, particularly when considering Grade Point Average (GPA) [13]. When academic measures such as Scholastic Assessment Test (SAT) scores are examined, students who spend more time gaming often show lower academic outcomes [13]. This pattern is not unique to a single country. A large meta-analysis combining data from more than 124,000 students across 28 countries reported a small but consistent decline in academic performance among individuals who spent more time playing video games [14]. Understanding this pattern may require examining daily habits, particularly sleep. One possible explanation is that longer gaming hours may be associated with shorter sleep duration; when students stay up late gaming, insufficient rest may in turn be associated with poorer classroom focus, memory, and overall academic functioning [15]. Some groups may be more vulnerable than others. For example, research suggests that male university students are more likely to show problematic gaming, which has been linked to lower college GPAs even when prior academic performance was strong [16]. Students who struggle with self-control may also be more prone to prolonged gaming sessions, potentially creating a cycle that affects academic outcomes [17]. Local findings echo these concerns: studies among secondary school students in Abha reported that frequent cybercafe gaming was associated with poorer academic performance, suggesting that certain gaming environments may intensify the problem [11,16].

A cross-sectional survey among medical students reported that problematic gaming was associated with poorer sleep quality and longer sleep latency (n = 356; mean age = 22.5 ± 1.8 years; males = 75.3%) [11]. A systematic review of 26 studies reported that video gaming was associated with reduced sleep quality and longer time to fall asleep, with effects varying by game type and time spent gaming [18]. In an online survey of 270 participants (mean age = 24.40 ± 6.98 years), two sleep profiles were identified (high sleep quality: n = 132; low sleep quality: n = 85), suggesting that sleep quality was related to gaming and mental health [19]. Another study using face-to-face interviews with adults (n = 844; 56.2% women; aged 18–94 years) reported that higher gaming volume was associated with poorer sleep quality, potentially due to prolonged screen exposure [20]. Experimental evidence among adolescents (n = 17; mean age = 16 ± 1 years) indicated that longer exposure to a violent video game (150 min vs. 50 min) was associated with poorer objective sleep quality compared with a neutral condition [21]. In a study of secondary school students in northern Turkey (n = 545), longer gaming time was associated with poorer sleep quality and lower competence [22]. Among Hong Kong university students (n = 300; mean (SD) age = 20.89; males = 122), IGDS9-SF scores showed a weaker association with sleep quality compared with social media addiction (BSMAS), and both IGD and SMA were associated with greater psychological problems and poorer sleep quality [23].

Although studies from Saudi Arabia and other countries have examined problematic gaming, sleep quality, and related mental health correlates, the specific combination of daily gaming time, sleep quality, and IGD symptom count has been less frequently examined within a single analytic model that includes both high school and university students in Riyadh. In particular, evidence remains limited regarding whether gaming time and sleep quality are independently associated with IGD symptom burden within the same student sample. Unlike passive screen time, video gaming is immersive, interactive, and often time-consuming, which may exert a stronger influence on sleep timing and sleep quality. This study therefore aimed to examine the associations between gaming time, sleep quality, and IGD symptom count among high school and university students in Saudi Arabia.

## 2. Methods and Materials

### 2.1. Study Design and Setting

This cross-sectional online survey was conducted in Riyadh, Saudi Arabia, between 10 September and 1 December 2025. The study was coordinated by Imam Mohammad Ibn Saud Islamic University and used a non-probability convenience sampling approach. The survey was distributed across multiple educational settings in Riyadh, including high schools and universities, through institutional and online dissemination channels. Because recruitment occurred through broad open distribution rather than a defined list of eligible students, no formal sampling frame was available, and the sample should not be considered representative of all students in Riyadh or Saudi Arabia.

### 2.2. Study Participants

A total of (N = 534) individuals were invited to participate. Of these, (n = 525) provided consent and (n = 9) did not consent. Among consenting participants, (n = 408) reported gaming, whereas (n = 117) reported not gaming and therefore had structural missingness on IGD variables due to skip logic. Analyses of IGD symptoms were restricted to gamers, with (n = 407) having complete IGD symptom count data. Sleep quality analyses using PSQI total score were restricted to participants with complete PSQI total score data (n = 352), meaning that (56) gamers had incomplete data required to derive the PSQI total score. For the adjusted negative binomial regression models, analyses were restricted to participants with complete data on all variables included in Model 2 (complete-case analysis; n = 347), indicating that an additional (5) participants with available PSQI total scores were excluded from the fully adjusted model because of missing covariate data. Differences in denominators across analyses reflect skip logic (non-gamers) and variable-specific missingness handled via complete-case analysis within each analysis/model.

### 2.3. Sample Size Calculation

We used the Raosoft sample size calculator to estimate the minimum sample required for the survey based on an estimated student population in Riyadh (approximately 1.46 million high school students and over 130,000 university students). Using a 95% confidence level and a 5% margin of error (with a conservative response distribution of 50%), the required minimum sample size was 385. The achieved consented sample (N = 525) and gamer subsample (n = 408) exceeded this minimum. However, the fully adjusted complete-case analytic sample (N = 347) was slightly below this survey-based target because participants with missing covariate or PSQI data could not be included in Model 2. Accordingly, this calculation should be interpreted as a pragmatic survey-planning rationale rather than a model-specific power analysis for the fully adjusted regression models.

### 2.4. Inclusion and Exclusion Criteria

Clear eligibility criteria were defined a priori and applied before entry into the analytic subsamples. The inclusion and exclusion criteria are summarized in Table 1.

### 2.5. Study Questionnaire and Data Collection

Data were collected using an online survey developed in Arabic and English and administered via Google Forms (Google LLC., Mountain View, CA, USA). The survey link was disseminated through educational networks in Riyadh, including invitations distributed to multiple schools and universities and through messaging platforms, including WhatsApp (Meta Platforms, Menlo Park, CA, USA) and Telegram (Telegram Messenger LLP, Dubai, United Arab Emirates). This approach was intended to reach students from more than one educational setting rather than from a single institution. Before accessing the questionnaire, participants viewed an information page describing the study purpose, voluntary participation, and confidentiality. Age screening was completed before access to the main questionnaire. For participants younger than 18 years, the survey was distributed through school-mediated channels and directed to parents/legal guardians. Those identified as minors were redirected to a parent/legal guardian information and consent page and were not allowed to proceed unless parental or legal guardian consent was electronically confirmed within the survey workflow before participation. Participants aged 18 years and older provided electronic informed consent before proceeding. These procedures were conducted in accordance with the IRB-approved protocol and national and institutional ethical requirements for research involving minors. After completion of the applicable consent process, participants completed a screening item indicating whether they played electronic games. Participants who answered this item affirmatively were classified as gamers for the purposes of the study. Non-gamers exited the IGD module due to skip logic, resulting in structural missingness for IGD variables, whereas gamers continued to the main questionnaire sections. Because the survey was distributed through broad open channels rather than to a closed, enumerated list of eligible students, a precise response rate could not be calculated. This open online recruitment strategy may also have introduced self-selection bias, potentially overrepresenting students with greater interest in gaming or related sleep concerns. The survey was divided into three parts.

#### 2.5.1. Participant Demographics and Background Data

This section collected key participant characteristics, including age group, gender, marital status, academic stage, academic-performance score on a common 0–100 scale, daily gaming time, and self-reported sleep duration over the past month. For university students, this value reflected GPA-equivalent academic performance, whereas for high school students it reflected school grades.

#### 2.5.2. Internet Gaming Disorder Indicators

IGD symptoms were assessed using the Arabic version of the Internet Gaming Disorder Scale (short form), a 9-item dichotomous (Yes/No) checklist derived from the (DSM-5) proposed 9 criteria for Internet Gaming Disorder. The 9 items map onto the (DSM-5) criteria (e.g., preoccupation, withdrawal, tolerance, unsuccessful attempts to control, giving up other activities, continued use despite problems, deception, gaming to escape negative mood, and jeopardized relationships/education). A symptom count (0–9) was computed by summing endorsed items, with higher scores indicating greater IGD symptom burden. The Arabic version followed a standard adaptation process, including permission from the original author, forward translation, back-translation, and expert review to reconcile discrepancies, with additional bilingual administration to ensure consistency between the Arabic and English versions. The validation study reported satisfactory Arabic/English validity and appropriate internal consistency and reliability. In the current sample, the nine IGD symptom items showed acceptable internal consistency (Cronbach’s α = 0.773). For descriptive purposes only, probable IGD was defined as endorsing (≥5) of the 9 criteria within a 12-month period, consistent with the (DSM-5) framework (24). This descriptive categorization was based on symptom count alone and should not be interpreted as a clinical diagnosis or as a substitute for clinical assessment of functional impairment or distress [24].

#### 2.5.3. Sleep Quality Assessment

Sleep quality over the past month was assessed using the Pittsburgh Sleep Quality Index (PSQI). The PSQI yields seven component scores—subjective sleep quality, sleep latency, sleep duration, habitual sleep efficiency, sleep disturbances, use of sleep medication, and daytime dysfunction—each scored (0–3), which are summed to produce a global PSQI score ranging from (0–21), with higher scores indicating poorer sleep quality. Following standard PSQI scoring, poor sleep was defined as (PSQI > 5) for descriptive purposes. Participants with missing information required to compute habitual sleep efficiency and/or the global PSQI score were treated as missing for these outcomes, resulting in a reduced denominator for analyses involving the PSQI total score. Most missingness affecting the PSQI total score arose from incomplete bedtime and/or wake-time information needed to derive habitual sleep efficiency and the global PSQI score. The Arabic version of the PSQI has demonstrated acceptable psychometric performance in a Saudi sample of medical students and interns, with moderate internal consistency overall (Cronbach’s alpha values ≥ 0.50 for the global score and several components, although subjective sleep quality and sleep latency showed lower alpha values <0.50) and moderate test–retest reliability over a 2-week interval (ICC ≈ 0.711 for the scale; global ICC = 0.694; component ICCs ranged from 0.460 to 0.763), with stable sleep-quality categories over time [25]. In the current sample, the seven PSQI components showed acceptable internal consistency (Cronbach’s α = 0.700).

### 2.6. Statistical Analysis

All analyses were conducted using IBM SPSS Statistics for Windows, Version 27.0 (IBM Corp., Armonk, NY, USA). Descriptive statistics summarized the gamer subsample, reporting categorical variables as frequencies and percentages and continuous variables (e.g., academic-performance score) as mean (SD) and range based on available data. For regression analyses, daily gaming time was modeled in ordered categories (<2, 2–4, 4–6, and ≥6 h/day) to reflect increasing levels of gaming exposure while preserving adequate cell sizes in the higher-duration groups; specifically, the 6–8 h/day and >8 h/day categories were combined into a single ≥6 h/day category because of the relatively small number of participants in these groups. Internet Gaming Disorder (IGD) severity was operationalized as an IGD symptom count (0–9) (treated as a count outcome). Sleep quality was assessed using PSQI component scores and the PSQI total score; poor sleep prevalence was described using (PSQI > 5) for descriptive purposes only.

Univariable associations between IGD symptom count and participant characteristics were examined using unadjusted negative binomial regression with a log link, reporting incidence rate ratios (IRR = Exp(B)) and 95% confidence intervals. In these models, IRRs and their corresponding confidence intervals were interpreted as effect-size estimates of association. Multivariable associations were examined using adjusted negative binomial regression: Model 1 included age group, gender, marital status, academic stage, daily gaming time, and academic-performance score; Model 2 additionally included PSQI total score. Categorical predictors used indicator coding with pre-specified reference categories (23+ years for age, female for gender, single for marital status, upper university years for academic stage, and ≥6 h/day for daily gaming time), and continuous predictors (academic-performance score and PSQI total score) were modeled per 1-point increase. These reference categories were selected to provide a consistent comparison framework across the ordered demographic and exposure variables and to allow interpretation of lower-exposure or earlier-stage groups relative to the highest gaming-time category and the most advanced academic stage. Model fit was evaluated using the likelihood ratio (LR) omnibus test and information criteria (AIC). Given that the primary multivariable analysis used negative binomial regression in an observational cross-sectional dataset, inference focused on incidence rate ratios and their 95% confidence intervals rather than on retrospective observed-power calculations.

Analyses used complete-case data within each model because the primary source of missingness involved incomplete bedtime and/or wake-time information required to derive the PSQI total score, and retaining complete cases within each model provided a transparent and internally consistent basis for estimation. Therefore, denominators differed across models due to missing data required to compute the PSQI total score and, in the fully adjusted model, a small amount of additional covariate missingness. We did not perform formal diagnostic testing to distinguish whether missingness was MCAR, MAR, or MNAR. Accordingly, complete-case results should be interpreted cautiously, because bias cannot be excluded if missingness was systematically related to observed or unobserved participant characteristics. All tests were two-tailed and statistical significance was set at (*p* < 0.05).

### 2.7. Ethical Approval

The study was reviewed and approved by the Institutional Review Board of Imam Mohammad Ibn Saud Islamic University (IMSIU) (protocol code: 857/2025; approval date: 10 September 2025). The approved study title was “Internet Gaming Disorder and Its Associated Factors Among High School and University Students in Riyadh City: A Cross-Sectional Study”. Informed consent was obtained from all participants aged 18 years and older. For participants younger than 18 years, age screening was completed before access to the main questionnaire, and parental or legal guardian consent was electronically confirmed within the survey workflow before participation. These procedures were conducted in accordance with the IRB-approved protocol and national and institutional ethical requirements for research involving minors.

## 3. Results

A total of (N = 534) individuals were invited to participate. Of these, (n = 525) provided consent and (n = 9) did not consent. Among consenting participants, (n = 408) reported gaming, whereas (n = 117) reported not gaming and therefore had structural missingness on Internet Gaming Disorder (IGD) variables due to skip logic. Table 2 shows the sociodemographic and gaming-related characteristics of the gamer subsample (N = 408). Most participants were aged 18–22 years (56.9%), followed by 15–17 years (34.1%), with relatively small proportions aged 23–25 years (4.2%) or older than 25 years (4.9%). The gender distribution was approximately balanced (49.5% male; 50.5% female), and the majority were single (95.8%). The high school students constituted 41.7% of the sample, while the remaining participants were distributed across university years, with the largest proportions in the first (17.6%), second (16.2%), and third (13.5%) university years. Regarding daily gaming time, half of the sample reported playing less than 2 h/day (50.2%), whereas smaller proportions reported 2–4 h/day (30.9%), 4–6 h/day (12.7%), 6–8 h/day (2.5%), or more than 8 h/day (3.7%). For sleep duration during the past month, most participants reported sleeping 6–8 h (37.5%) or 8–10 h (30.4%), while 18.9% reported 4–6 h and 2.2% reported less than 4 h. Using available data, the mean academic-performance score (0–100) was 89.80 (SD = 14.88; range = 14–100; n = 402). The observed proportion of gamers in this convenience sample should not be interpreted as a population prevalence estimate for students in Riyadh.

Table 3 shows the distribution of IGD symptom counts (0–9) and the prevalence of probable IGD among gamers with complete IGD data (n = 407). Symptom counts were concentrated in the low-to-moderate range, with the most common values being 1 symptom (17.0%), 2 symptoms (19.2%), and 3 symptoms (15.2%). Overall, 25.3% of gamers met the descriptive criterion for probable IGD by endorsing ≥5 of the 9 DSM-5-based criteria, whereas 74.7% did not. Among those meeting this descriptive threshold, symptom counts of 5 (9.8%), 6 (6.1%), 7 (5.9%), and 8 (3.4%) were observed. This symptom-count categorization was used for descriptive purposes only and should not be interpreted as a clinical diagnosis of Internet Gaming Disorder.

Table 4 summarizes sleep quality among gamers using the Pittsburgh Sleep Quality Index (PSQI). Among participants with complete PSQI total scores (n = 352), the mean PSQI total score was 7.49 (SD = 3.46; range = 1–21), indicating poor sleep quality on average. Poor sleep (PSQI > 5) was observed in 68.2% (240/352), whereas 31.8% (112/352) had PSQI scores ≤ 5. At the component level (0–3), the highest mean scores were observed for habitual sleep efficiency (M = 1.56, SD = 1.33; n = 352) and sleep latency (M = 1.33, SD = 0.93; n = 407), followed by daytime dysfunction (M = 1.17, SD = 0.87; n = 407) and sleep disturbances (M = 1.13, SD = 0.56; n = 407); use of sleep medication was relatively low (M = 0.33, SD = 0.71; n = 407). Differences in denominators reflect missing bedtime/wake-time information required to compute habitual sleep efficiency and the PSQI total score.

Table 5 presents unadjusted negative binomial regression models estimating incidence rate ratios (IRRs) for IGD symptom count (0–9) among gamers. Daily gaming time was strongly associated with symptom count: compared with participants playing ≥6 h/day, those playing <2 h/day had a significantly lower expected count (IRR = 0.527, 95% CI = 0.405–0.686, *p* < 0.001), and those playing 2–4 h/day also had a lower expected count (IRR = 0.694, 95% CI = 0.530–0.910, *p* = 0.008), whereas the 4–6 h/day group did not differ significantly (IRR = 0.879, 95% CI = 0.652–1.183, *p* = 0.394). Poor sleep quality (PSQI > 5) was associated with a higher expected symptom count (IRR = 1.402, 95% CI = 1.183–1.661, *p* < 0.001). Age group was also associated with symptom count, with both the 15–17 years (IRR = 0.744, 95% CI = 0.576–0.961, *p* = 0.023) and 18–22 years (IRR = 0.783, 95% CI = 0.614–0.998, *p* = 0.048) groups showing lower expected symptom counts relative to the 23+ years group. No statistically significant differences were observed for gender, marital status, or academic stage (ps > 0.05). As noted, analyses were restricted to gamers with complete IGD outcome data (n = 407), while the poor sleep model was restricted to participants with complete PSQI poor-sleep classification data (n = 352).

Table 6 presents adjusted incidence rate ratios (IRRs) from negative binomial regression models predicting IGD symptom count (0–9) among gamers. In Model 1, daily gaming time was the only factor significantly associated with symptom count after adjustment for age group, gender, marital status, academic stage, and academic-performance score. Compared with participants playing ≥6 h/day, those playing <2 h/day had a significantly lower expected symptom count (Adjusted IRR = 0.531, 95% CI = 0.392–0.718, *p* < 0.001), and those playing 2–4 h/day also had a lower expected count (Adjusted IRR = 0.675, 95% CI = 0.497–0.917, *p* = 0.012), whereas the 4–6 h/day group did not differ significantly (Adjusted IRR = 0.955, 95% CI = 0.683–1.337, *p* = 0.790). In Model 2, after additionally adjusting for PSQI total score, higher PSQI total score was significantly associated with a higher expected symptom count (Adjusted IRR = 1.049 per 1-point increase, 95% CI = 1.029–1.071, *p* < 0.001), while daily gaming time remained significant (<2 h/day: Adjusted IRR = 0.584, *p* < 0.001; 2–4 h/day: Adjusted IRR = 0.720, *p* = 0.029). No statistically significant adjusted associations were observed for age group, gender, marital status, academic stage, or academic-performance score in either model (*p* > 0.05).

## 4. Discussion

The widespread use of video games as a form of entertainment among young people worldwide has become increasingly evident. Numerous studies have suggested that a substantial number of children and adolescents spend a significant portion of their awake hours engaging with electronic entertainment, such as video games [26]. Although video gaming is frequently perceived as an enjoyable and stimulating leisure activity by the majority of individuals, it can develop into a highly addictive and even debilitating behavior for certain individuals [27]. Conceptually, Internet Gaming Disorder and prolonged gaming sessions may be associated with sleep difficulties through behavioral displacement of bedtime, increased cognitive and emotional arousal, and disruption of healthy sleep routines in high school and university students. Poor sleep quality, in turn, may be associated with reduced concentration, poorer academic functioning, lower well-being, and diminished quality of life. Heavy immersion in virtual environments may also coincide with lower physical activity, reduced face-to-face social engagement, and less consistent daily routines. In this context, insomnia, headache, and pain complaints may co-occur with poor sleep and further compromise daytime functioning and perceived quality of life [28,29].

This study aimed to examine the associations between gaming time, sleep quality, and IGD symptom count among high school and university students in Saudi Arabia.

The study used a cross-sectional, online survey with a non-probability convenience sampling strategy to recruit high school and university students in Riyadh through educational networks, including schools, universities, and messaging platforms such as WhatsApp and Telegram. Although the achieved sample size (N = 408 consenting participants) exceeded the minimum calculated requirement of 385, the findings should still be interpreted cautiously because the study used a non-probability convenience sample and was not designed to yield a representative prevalence estimate. In addition, recruitment through educational networks and open online dissemination channels may have increased the likelihood of self-selection by students with greater interest in gaming or related sleep concerns.

There were (N = 408) gamers in the analytic sample. Gender was approximately balanced, consistent with prior studies from Saudi Arabia [30,31] and contrasts with a global systematic review and meta-analysis suggesting higher IGD among males [32]. Most participants were adolescents and young adults, and the majority were single, consistent with other studies in Saudi Arabia [30,31]. High school students constituted a substantial proportion of the sample, and the remainder were distributed across university years. About half of participants reported playing video games for less than two hours per day. Although many participants reported sleeping (6–10) hours per night, PSQI results indicated a high prevalence of poor sleep quality among those with complete PSQI total scores.

IGD symptom counts were concentrated in the low-to-moderate range; however, the observed prevalence of probable IGD (25.3%) was higher than that reported in some meta-analytic and regional studies [31,33,34]. This comparatively high proportion should be interpreted cautiously because the study used a convenience sample of self-identified gamers, relied on a dichotomous symptom checklist, and was not designed to estimate population prevalence among all students in Riyadh.

Adequate sleep is important for daily functioning, including attention, memory, and behavior [35,36]. Prior research suggests that greater screen time may be linked to shorter sleep duration and poorer sleep quality, which in turn may relate to academic outcomes. For example, a study of 3095 Spanish adolescents (aged (12–18)) reported that higher academic performance was associated with less screen time and more sleep hours [37]. Similarly, a study of 269 adolescents aged (14) years found that poorer sleep quality was associated with lower academic performance [38].

Higher IGD symptom counts were associated with longer daily gaming time and poorer sleep quality. In contrast, no statistically significant adjusted associations were observed for age group, gender, marital status, or academic stage in the adjusted models.

In this cross-sectional sample, longer daily gaming time was associated with higher IGD symptom counts. Compared with participants playing ≥6 h/day, those reporting fewer gaming hours had lower expected symptom counts. This pattern is consistent with a study conducted among high school and middle school students in Albaha, Saudi Arabia [6]. It is also consistent with findings reported among college students in Saudi Arabia [39], suggesting that reduced gaming time is associated with lower IGD symptom burden.

Sleep quality was additionally included in the adjusted model to examine whether it was associated with IGD symptom count beyond demographic variables, daily gaming time, and academic-performance score. Higher PSQI total scores (indicating poorer sleep quality) remained significantly associated with higher expected IGD symptom counts. This finding is consistent with evidence reported among college students in Jordan [40]. However, given the cross-sectional design, the direction of this association remains unclear: poorer sleep may be linked to greater IGD symptom burden, greater IGD symptom burden may be linked to poorer sleep, or both may reflect shared underlying factors. In addition, unmeasured factors such as depression, anxiety, broader psychological distress, academic stress, caffeine intake, physical activity, family environment, and other screen-based behaviors may plausibly contribute to both poorer sleep and greater gaming-related problems.

In the adjusted models, age group, gender, marital status, academic stage, and academic-performance score were not significantly associated with IGD symptom count. Several explanations are possible. First, the reduced complete-case sample for the adjusted analyses may have limited precision for detecting smaller associations. Second, some of these associations may have been attenuated after simultaneous adjustment for stronger correlates, particularly daily gaming time and sleep quality. Third, the academic-performance indicator combined school grades and university GPA on a common 0–100 scale, which may have introduced measurement heterogeneity and reduced comparability across educational stages. Accordingly, these null findings should not be interpreted as definitive evidence of no association. This pattern contrasts with a study among students at Imam Mohammad Ibn Saud Islamic University reporting higher IGD among males and an association with lower GPA [41], and it also differs from studies in China and Norway that reported higher IGD among males [42,43]. Similarly, a study from India also reported higher IGD among males [44].

Overall, poorer sleep quality and longer daily gaming time were the strongest correlates of higher IGD symptom counts in this sample.

## 5. Study Limitations and Future Research

This study has several limitations. First, data were collected using self-administered questionnaires, which may be subject to self-report bias and recall bias. Future studies could incorporate objective or multi-method assessments, such as actigraphy, device-based sleep monitoring, gaming logs, or interviews, to strengthen measurement accuracy. Second, participants were recruited using a non-probability online convenience sample through multiple educational settings and online dissemination channels in Riyadh. Because the survey was distributed through broad open channels without a defined sampling frame, representativeness could not be formally assessed and a conventional response rate could not be calculated. This recruitment strategy may have introduced self-selection bias, potentially overrepresenting students who were more interested in gaming or had greater sleep-related concerns. Accordingly, the findings should be generalized cautiously and should not be assumed to represent all students in Riyadh or all Saudi students. Future research should use probability-based or institutionally enumerated sampling strategies where feasible. Third, the cross-sectional design precludes causal inference and does not permit conclusions about temporal directionality; longitudinal studies are needed to determine whether gaming behavior precedes sleep problems and greater IGD symptom burden, whether sleep problems precede greater IGD symptom burden, or whether these associations are bidirectional. Fourth, we did not assess several potentially relevant factors that may help explain the observed associations, including depression, anxiety, broader psychological distress, coping motives, social motivations, academic stress, caffeine intake, physical activity, family environment, and other screen-based behaviors. Moreover, the academic-performance measure combined school grades and university GPA on a common 0–100 scale, which may have introduced measurement heterogeneity across educational stages. Future studies may benefit from stage-specific standardization strategies when comparing academic performance across educational levels. These unmeasured variables may be related to both gaming behavior and sleep quality and could partly account for some of the associations observed in this study, resulting in residual confounding. Finally, analyses used complete-case data within each model, and denominators differed across analyses due to variable-specific missingness. In particular, (56) gamers had incomplete data required to derive the PSQI total score, and an additional (5) participants were excluded from the fully adjusted model because of missing covariate data. We did not formally test whether the missingness mechanism was MCAR, MAR, or MNAR; therefore, although complete-case analysis provided a transparent and consistent analytic approach, some bias cannot be excluded if missingness was systematically related to participant characteristics or outcome severity. Future studies should consider prospectively minimizing item nonresponse and, where appropriate, using justified missing-data methods and sensitivity analyses.

## 6. Conclusions

Most gamers reported relatively short daily gaming periods, although a smaller proportion reported longer gaming durations. IGD symptom counts were concentrated in the low-to-moderate range, and poor sleep quality was prevalent among participants with complete PSQI total scores (PSQI > 5: 68.2%). In adjusted models, longer daily gaming time was significantly associated with higher IGD symptom counts, and higher PSQI total score was significantly associated with higher symptom counts. These findings highlight sleep quality and gaming time as potentially relevant correlates for prevention and intervention efforts among adolescents and young adults; however, longitudinal studies are needed to clarify temporal directionality and potential causal pathways.

## Figures and Tables

**Table 1:** Summary of inclusion and exclusion criteria.

Inclusion Criteria	Exclusion Criteria
High school or university students	Non-residents of Riyadh
Aged 15 years or older, consistent with the usual age at entry into high school in Saudi Arabia	Younger than 15 years
Able to understand Arabic or English	Unable to understand the questionnaire language
Provided consent to participate	Did not provide consent
Reported playing electronic games	Non-gamers were excluded from IGD analyses because of skip logic

**Table 2:** Participant characteristics (N = 408).

Characteristic	n	%
Age group		
15–17 years	139	34.1%
18–22 years	232	56.9%
23–25 years	17	4.2%
Older than 25 years	20	4.9%
Gender		
Male	202	49.5%
Female	206	50.5%
Marital status		
Single	391	95.8%
Married	17	4.2%
Academic stage		
First year of university (or preparatory year)	72	17.6%
Second year of university	66	16.2%
Third year of university	55	13.5%
Fourth year university	19	4.7%
Fifth year of university	13	3.2%
Sixth year university	5	1.2%
Seventh year of university (excellence)	8	2.0%
High school	170	41.7%
Daily gaming time		
Less than 2 h/day	205	50.2%
2–4 h/day	126	30.9%
4–6 h/day	52	12.7%
6–8 h/day	10	2.5%
More than 8 h/day	15	3.7%
Sleep duration (last month)		
Less than 4 h	9	2.2%
4–6 h	77	18.9%
6–8 h	153	37.5%
8–10 h	124	30.4%
10–12 h	45	11.0%
Academic-performance score (0–100), mean (SD)	402	89.80 (14.88)

Note. Percentages are based on the gamer subsample (N = 408) and may not sum to 100 due to rounding. Academic-performance score is reported using available data (n = 402).

**Table 3:** Internet Gaming Disorder (IGD) symptom count distribution and probable IGD prevalence among gamers (N = 407).

IGD Indicator	n	%
IGD symptom count (0–9)		
0	47	11.5%
1	69	17.0%
2	78	19.2%
3	62	15.2%
4	48	11.8%
5	40	9.8%
6	25	6.1%
7	24	5.9%
8	14	3.4%
9	0	0.0%
Total	407	100%
Probable IGD (≥5/9)		
No probable IGD (<5/9)	304	74.7%
Probable IGD (≥5/9)	103	25.3%

Note. Results are based on gamers with complete IGD data (n = 407); one participant had missing IGD data.

**Table 4:** Sleep quality measured by the Pittsburgh Sleep Quality Index (PSQI) among gamers.

PSQI Domain/Indicator	n	Mean	SD	Min–Max
PSQI components (0–3)				
Subjective sleep quality	407	1.10	0.81	0–3
Sleep latency	407	1.33	0.93	0–3
Sleep duration	407	0.99	1.04	0–3
Habitual sleep efficiency	352	1.56	1.33	0–3
Sleep disturbances	407	1.13	0.56	0–3
Use of sleep medication	407	0.33	0.71	0–3
Daytime dysfunction	407	1.17	0.87	0–3
PSQI total score (0–21)	352	7.49	3.46	1–21
Poor sleep (PSQI > 5)				
No (≤5)	112	31.8%		
Yes (>5)	240	68.2%		

Note. PSQI = Pittsburgh Sleep Quality Index; higher scores indicate worse sleep quality. Component scores range from 0–3 and total score from 0–21. Poor sleep was defined as PSQI total score >5. The PSQI total score and habitual sleep efficiency were available for a subsample (n = 352) because 56 gamers had missing bedtime and/or wake-time information required for their calculation; other component scores were available for (n = 407). Percentages for poor sleep are based on (n = 352).

**Table 5:** Unadjusted negative binomial models (IRR) for IGD symptom count (0–9) among gamers.

Factor	Category (n)	IRR	95% CI for IRR	*p*
Age group (ref = 23+ years)				
15–17 years	139	0.744	0.576–0.961	0.023
18–22 years	231	0.783	0.614–0.998	0.048
23+ years	37	Ref	—	—
Gender (ref = Female)				
Male	201	1.043	0.903–1.206	0.566
Female	206	Ref	—	—
Marital status (ref = Single)				
Married	17	1.194	0.845–1.686	0.314
Single	390	Ref	—	—
Academic stage (ref = Upper university years 4th–7th)				
High school	170	0.819	0.646–1.039	0.100
Lower university years (1st–3rd)	192	0.881	0.698–1.112	0.286
Upper university years (4th–7th)	45	Ref	—	—
Daily gaming time (ref = ≥6 h/day)				
Less than 2 h/day	204	0.527	0.405–0.686	<0.001
2–4 h/day	126	0.694	0.530–0.910	0.008
4–6 h/day	52	0.879	0.652–1.183	0.394
≥6 h/day	25	Ref	—	—
Poor sleep quality (PSQI > 5) (ref = No)				
Yes (>5)	240	1.402	1.183–1.661	<0.001
No (≤5)	112	Ref	—	—

Note. Analyses were restricted to gamers with complete IGD outcome data (n = 407). The poor sleep model was restricted to participants with complete PSQI poor-sleep classification data (n = 352). Reference categories: age (23+ years), gender (female), marital status (single), academic stage (upper university years), daily gaming time (≥6 h/day), and sleep quality (no poor sleep).

**Table 6:** Adjusted incidence rate ratios (IRRs) for Internet Gaming Disorder symptom count using negative binomial regression (MLE) (N = 347).

Predictor	Category/Contrast	Model 1 Adjusted		Model 2 Adjusted	
		IRR (95% CI)	*p*	IRR (95% CI)	*p*
Age group	15–17 vs. 23+ (reference category)	0.884 (0.601–1.299)	0.530	0.961 (0.660–1.399)	0.834
	18–22 vs. 23+ (reference category)	0.774 (0.554–1.082)	0.134	0.836 (0.604–1.157)	0.281
Gender	Male vs. Female (reference category)	0.989 (0.843–1.162)	0.896	1.010 (0.865–1.180)	0.897
Marital status	Married vs. Single (reference category)	1.001 (0.673–1.488)	0.997	0.951 (0.647–1.399)	0.799
Academic stage	High school vs. Upper university (reference category)	0.748 (0.528–1.058)	0.101	0.772 (0.551–1.083)	0.134
	Lower university vs. Upper university (reference category)	0.928 (0.686–1.256)	0.630	0.945 (0.705–1.267)	0.705
Daily gaming time	<2 h/day vs. ≥6 h/day (reference category)	0.531 (0.392–0.718)	<0.001	0.584 (0.435–0.783)	<0.001
	2–4 h/day vs. ≥6 h/day (reference category)	0.675 (0.497–0.917)	0.012	0.720 (0.536–0.967)	0.029
	4–6 h/day vs. ≥6 h/day (reference category)	0.955 (0.683–1.337)	0.790	0.989 (0.717–1.366)	0.949
Academic-performance score (0–100)	Per 1-point increase	0.999 (0.994–1.004)	0.656	0.999 (0.994–1.003)	0.592
Sleep quality (PSQI total)	Per 1-point increase	—	—	1.049 (1.029–1.071)	<0.001

Note. Analyses were restricted to participants with complete data for all variables included in Model 2 (complete-case analysis; N = 347); Model 1 was estimated on the same analytic sample for comparability. Outcome is IGD symptom count. Model 1 includes age group, gender, marital status, academic stage, daily gaming time, and academic-performance score. Model 2 includes Model 1 covariates plus PSQI total score. Reference categories were 23+ years (age), female (gender), single (marital status), upper university years (academic stage), and ≥6 h/day (daily gaming time). Results are reported as adjusted IRR = Exp(B) with 95% Wald confidence intervals; *p*-values are two-tailed.

## Data Availability

The datasets generated and analysed during the current study are not publicly available due to participant privacy and ethical restrictions associated with the approved protocol but are available from the corresponding author on reasonable request. De-identified data and the statistical analysis materials can be shared in accordance with institutional requirements and applicable approvals.
